# Exposure to Endocrine Disrupting Chemicals and Risk of Breast Cancer

**DOI:** 10.3390/ijms21239139

**Published:** 2020-11-30

**Authors:** Louisane Eve, Béatrice Fervers, Muriel Le Romancer, Nelly Etienne-Selloum

**Affiliations:** 1 Faculté de Pharmacie, Université de Strasbourg, F-67000 Strasbourg, France; louisane.eve@etu.univ-lyon1.fr; 2 Université Claude Bernard Lyon 1, F-69000 Lyon, France; 3 Inserm U1052, Centre de Recherche en Cancérologie de Lyon, F-69000 Lyon, France; 4 CNRS UMR5286, Centre de Recherche en Cancérologie de Lyon, F-69000 Lyon, France; 5 Centre de Lutte Contre le Cancer Léon-Bérard, F-69000 Lyon, France; beatrice.fervers@lyon.unicancer.fr; 6 Inserm UA08, Radiations, Défense, Santé, Environnement, Center Léon Bérard, F-69000 Lyon, France; 7 Service de Pharmacie, Institut de Cancérologie Strasbourg Europe, F-67000 Strasbourg, France; 8 CNRS UMR7021/Unistra, Laboratoire de Bioimagerie et Pathologies, Faculté de Pharmacie, Université de Strasbourg, F-67000 Strasbourg, France

**Keywords:** breast cancer, endocrine disrupting chemicals, diethylstilbestrol, DDT, dioxin, bisphenol A

## Abstract

Breast cancer (BC) is the second most common cancer and the fifth deadliest in the world. Exposure to endocrine disrupting pollutants has been suggested to contribute to the increase in disease incidence. Indeed, a growing number of researchershave investigated the effects of widely used environmental chemicals with endocrine disrupting properties on BC development in experimental (in vitro and animal models) and epidemiological studies. The complex effects of endocrine disrupting chemicals (EDCs) on hormonal pathways, involving carcinogenic effects and an increase in mammary gland susceptibility to carcinogenesis—together with the specific characteristics of the mammary gland evolving over the course of life and the multifactorial etiology of BC—make the evaluation of these compounds a complex issue. Among the many EDCs suspected of increasing the risk of BC, strong evidence has only been provided for few EDCs including diethylstilbestrol, dichlorodiphenyltrichloroethane, dioxins and bisphenol A. However, given the ubiquitous nature and massive use of EDCs, it is essential to continue to assess their long-term health effects, particularly on carcinogenesis, to eradicate the worst of them and to sensitize the population to minimize their use.

## 1. Introduction

Breast cancer (BC) is the second most common and deadliest cancer in women worldwide, accounting for 24.2% of new cancer cases and 15% of deaths [1]. Despite the number and diversity of treatments available and increased patient survival, BC remains a major health issue.

According to the American Cancer Society, one in eight women (13%) will develop BC during her lifetime, and one in 39 women (3%) will die from it. Between 1980 and 2000, the incidence rate of BC increased rapidly [2]. This increase is multifactorial and may be explained in part by technological advances in mammography screening [3] and population aging, but also by changes in lifestyle such as diet, obesity and lack of physical activity, reproductive factors (age of puberty, age of first child, breastfeeding, age of menopause) or environmental exposures [4]. BC incidence then decreased in the early 2000s in women over 50 years of age, following a decrease in the prescription of hormone replacement therapy (HRT) [5]. Since then, its incidence rate has slowly been rising and the World Health Organization (WHO) predicts a 63.4% increase by 2040 [6]. Changes in lifestyle and environmental factors, as well as an aging population, will be responsible for most of this upsurge. According to the International Agency for Research on Cancer (IARC), 36.8% of BC are attributable to lifestyle and environmental factors in adults over 30 years of age. These risk factors include alcohol consumption (15.1%), high body mass index (BMI) (8.4%), smoking (4.4%), diet (4.3%) and lack of physical activity (3%) [7]. Approximately 13% of BC can be attributed to genetic factors [8]. In addition, high-dose and long-term exposure to endogenous estrogens is known to be a major risk factor for BC [9]. Exposure to certain exogenous estrogens such as HRT [10] or hormonal contraceptives increases the risk of developing BC [11]. Nevertheless, the impact of exposure to other exogenous estrogens such as phytoestrogens remains controversial [12]. In addition to changes in lifestyle and reproduction, exposure to environmental pollutants—in particular pollutants with endocrine disrupting properties—has been suggested to contribute to the increased incidence of BC [13].

Given the impact of agricultural and industrial chemicals on wildlife, some experts hypothesized that they may be involved in the growing incidence of cancers in humans. In this context, Theodora Colborn, an expert in zoology and epidemiology, organized the Wingspread conference in 1991. This conference provided a review on the release of synthetic chemicals into the environment and their potential effects on the endocrine systems of animals and humans. This conference ended with a consensus statement in which the 32 speakers proposed several ways to improve knowledge on agricultural and industrial chemicals in order to limit damage to the environment and human health. In the declaration, they called for changes in the regulations on the use of toxic substances and their release in the environment. According to them, it was necessary for the industry producing a chemical product to provide proof of its safety. They also called for a precautionary approach to be applied to a substance whose effects are not fully understand [14]. It was in this statement that the term endocrine disrupting chemicals (EDCs) was first used. Three years later, the first review on the underlying mechanisms of EDCs on wildlife—but also on humans—marks the beginning of the general interest in EDCs [15]. Since then, the concept of EDCs has been widely studied. An EDC is a compound that mimics, disrupts or antagonizes the pathways of the endocrine system. Among the hormones of the endocrine system are steroid hormones such as estradiol or testosterone that act on steroid receptors (SRs). SRs are transcription factors involved in various biological functions, and the binding of EDCs results in the dysfunction of reproduction, growth and development, sleep, etc. [16]. They may be carcinogenic, cause reproductive disorders, induce polycystic ovary syndrome, aneuploidy, premature ovarian failure, reproductive tract abnormalities, uterine fibrosis, endometriosis, and ectopic pregnancy [17].

Xenoestrogens are a type of EDC that mimics the activity of estradiol. Most often, they impact the estrogen pathway by binding to its receptors, modifying its biosynthesis or degradation, or activating its transcriptional activity [18]. Xenoestrogens can be synthetic or they can be natural, as in the case of phytoestrogens found mainly in soybeans [12]. The impact of xenoestrogens on the risk of BC has been studied because of the carcinogenic effect of estrogens on breast epithelial cells. The particular modes of action of EDCs make them difficult to study in vitro and in vivo, nevertheless, effects have been observed for some. In addition, epidemiological studies are difficult to carry out because EDCs are ubiquitously present in the environment at low dose, making the constitution of a control cohort almost impossible. For some EDCs, however, particular events have exposed a population to EDCs, making it possible to form a high exposure cohort compared with a low exposure cohort [13].

The objective of this review is to investigate the impact of exposure to EDCs on the risk of developing BC. First, the specificities of the mammary gland as well as the pathogenesis of BC and its various risk factors will be explained. Then, the characteristics of EDCs will be clarified in order to highlight the reasons why the categorization of their pathogenic effects is difficult. Finally, the case of four established EDCs will be studied: diethylstilbestrol (DES), dichlorodiphenyltrichloroethane (DDT), dioxins and in particular 2,3,7,8-tetrachlorodibenzo-p-dioxin (TCDD), and bisphenol A (BPA). For each, knowledge on their in vitro and in vivo mode of action, the various epidemiological studies on BC risk, and current regulations will be reviewed.

## 2. Breast Cancer, the Most Common Cancer in Women Sill on the Rise

### 2.1. Specificities of the Mammary Gland and Its Windows of Susceptibility

The mammary gland is an exocrine gland that evolves throughout a woman’s life. The first phase, in utero, is common to both women and men. At the end of embryonic development, the mammary gland consists of a short primary canal ending in a rudimentary ductal tree embedded in a fat pad. At puberty, the primary ducts branch into segmental and sub-segmental ducts in women under the effect of estrogen, progesterone and other growth hormones. The terminal end buds (TEBs) are located at the end of the growing intralobular ducts and allow for ductal lengthening and branching of the epithelial tree. The remaining space is invaded by fatty tissue, blood vessels, immune cells and fibroblasts. In men, increase in testosterone levels inhibits the development of the mammary gland. The mammary gland may go through two additional stages of development for some women during pregnancy and breastfeeding. Proliferation of epithelial cells generates alveolar buds that cleave and gradually differentiate into distinct alveoli, becoming lobules that secrete milk during lactation. The end of lactation is marked by involution of the mammary gland with remodeling of the basement membrane and collapse of the alveoli. Finally, the mammary gland goes through an age-induced involution during which the glandular epithelium and interlobular connective tissue are replaced by adipose tissue [19]. Thus, the mammary gland develops throughout a woman’s life with three to five phases of intense morphological changes and cell proliferation. These specific periods (including prenatal development, puberty, pregnancy, and menopausal transition) represent windows of increased susceptibility to environmental exposure that may increase the risk of BC [13,20].

### 2.2. Complementary Classifications of Breast Cancer

BC is a very heterogeneous pathology and different classifications exist to distinguish them. The histological classification separates in situ tumors, which proliferate without invading the basement membrane, from infiltrating tumors that invade the connective tissue. In situ carcinomas can be ductal or lobular. In situ ductal carcinomas represent 15–20% of BC and several subtypes exist (solid, papillary, micropapillary, cribriform, etc.). In situ lobular carcinomas correspond to only 0.5% of BC and are considered to be a precursor form of invasive cancer. Invasive cancers present a risk of lymph node invasion. The most common is invasive ductal carcinoma, which accounts for 70% of invasive BC. Many other types exist such as lobular carcinoma, tubular carcinoma, mucinous carcinoma, cystic adenoid or cylindroid carcinoma, cribriform carcinoma, and medullary carcinoma [21]. The molecular classification was carried out by analyzing approximately 500 genes per DNA chip [22]. It is based on the expression status of three main types of receptors: estrogen receptor α (ERα), progesterone receptor (PR) and human epidermal growth factor receptor 2 (HER2). The Ki proliferation index 67 (Ki67) is an additional prognostic marker. This classification distinguishes luminal A (ERα+ and/or PR+, HER2−, low Ki67), luminal B (ERα+ and/or PR+, HER2+/−, high Ki67), HER2 overexpressing cancers (ERα−, PR−, HER2+), and triple negative BC (ERα−, PR−, HER2−) (TNBC) [23]. These different subtypes display significant differences in terms of survival. The TNBC subgroup is very heterogeneous and can itself be classified into seven subtypes according to gene expression profiles: basal-like 1, basal-like 2, immunomodulatory, mesenchymal, mesenchymal stem-like, luminal androgen receptor (AR) and unstable [24].

These two classifications—the histological classification on the aggressiveness of the tumor and the molecular classification—are complementary, and their combination orients patient management and the use of targeted therapies. The majority of BC (75%) are ERα+ and/or PR+ and can be treated with hormonal therapy (aromatase inhibitors, ERα antagonists). Approximately 15% of BC overexpress HER2, allowing them to be targeted with anti-HER2 antibodies (such as trastuzumab and its derivatives, pertuzumab) or kinase inhibitors of this receptor (such as lapatinib, tucatinib, neratinib). According to the stage and aggressiveness of the disease, cytotoxic drugs may be added to hormonal and/or targeted therapies. For the 12% of TNBCs, cytotoxic drugs have long been the only treatments available [25,26]. However, some immunotherapies have been evaluated to treat TNBC in association with cytotoxic drugs. For instance, atezolizumab, an anti-PDL1 antibody, suppresses the inhibition of immune responses observed in some cancers [27]. 

### 2.3. A Complex Combination of Risk Factors

BC is a multifactorial disease, so its exact cause is usually unknown. There are many risk factors suspected to increase its occurrence, but the precise impact of each of them is not known. BC risk factors can be divided into different categories: reproductive factors, exogenous hormones, anthropometric factors, sex and age, breast density and personal history of BC, familial history of BC, lifestyle, occupation, and exposure to radiation (Table 1). 

Despite the importance of these established risk factors, they underlie only approximately 36.8% of BCs, arguing in favor of further studies on the role of environmental contaminants in BC risk [7]. In addition, certain risk factors are associated with specific histological and molecular subtypes. In fact, a meta-analysis of 38 studies unveiled that the majority of the established risk factors were mainly for the luminal A subtype [28].

## 3. Difficult Identification of the Mode of Action of Endocrine Disrupting Chemicals

### 3.1. A Recent Concept in Constant Evolution

Currently, there is no agreement on the exact definition of EDCs between the different health institutions. For both the Food and Drug Administration (FDA) and the WHO, EDCs are defined by their mechanisms of action rather than by their origin or structure. Nevertheless, the two agencies differ on their definition of the effects of EDCs. For the WHO, EDCs have an intrinsic deleterious effect [44]. Whereas, for the FDA they can modify the endocrine system without having a harmful effect [45]. This difference is crucial because if EDCs induce intrinsic deleterious effects, the regulations regarding their use should be stricter.

In 1991, the biologist Ana Soto proved for the first time that EDCs, such as nonylphenols, can have deleterious effects when present in materials used daily by the population [46]. This substance is considered toxic to aquatic life by the USA Environmental Protection Agency (EPA). However, nonylphenols are still used in several products including industrial and domestic cleaning products, cosmetics and personal hygiene products [47]. In other countries like France, nonylphenol is considered to be an EDC and is no longer used in the composition of these products [48].

The IARC is a WHO agency that studies agents and classifies them according to their carcinogenicity: carcinogenic, probably carcinogenic, possibly carcinogenic, and not classifiable as to its carcinogenicity on humans. To do this, the IARC reviews the epidemiological, experimental animal and in vitro studies published on these agents. The evaluation of these studies as to the plausibility of a causal link is based on Bradford Hill’s criteria since their publication in 1965 [49,50]. Hill’s criteria (strength of association, consistency, specificity, temporality, biological gradient, plausibility, coherence, experiment, and analogy) lead to attributing an effect to an agent [51]. Novel insights into cancer mechanisms, essential for carcinogen hazard identification, have led the IARC monographs to integrate an explicit approach for a holistic consideration of the mechanistic evidence of carcinogens [52].

Several challenges exist to apply the Hill criteria for the assessment of EDCs, particularly with regard to their varied modes of action, the existence of non-monotonic dose-response relationships, frequent exposures to EDC mixtures, effects of exposure during critical periods, and varying effects according to the hormonal status of the target organ. Very recently, a consensus statement on the key characteristics of EDCs was published [53]. It provides a basis for the search, organization and evaluation of mechanistic evidence for the identification of carcinogens.

### 3.2. Effects of Endocrine Disrupting Chemicals on Hormone Signaling

#### 3.2.1. Agonistic or Antagonistic Action on Different Hormone Receptors

**Figure 1:**
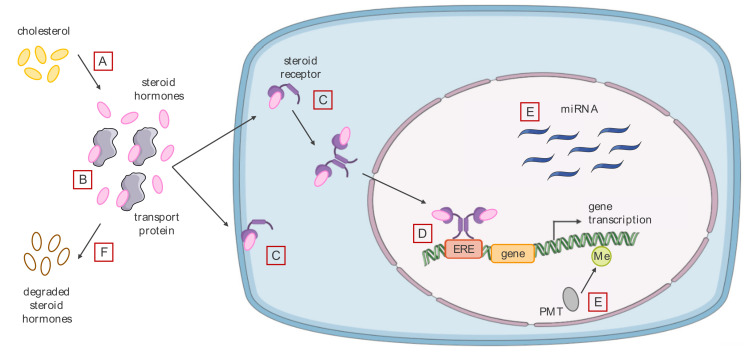
Key Characteristics of endocrine disrupting chemicals (EDCs). (**A**) EDCs can modulate the synthesis of steroid hormones from cholesterol by modulating the expression of the enzymes involved in the process. (**B**) They may also compete with steroid hormones for binding to transport proteins, thereby modulating the free fraction of steroid hormones. (**C**) EDCs can bind to steroid receptors (SRs) (estrogens, androgens, aryl hydrocarbon) and act as agonists or antagonists. (**D**) The SRs—EDCs complex can then bind to DNA via, for example, the estrogen response element (ERE) and thus modulate the expression of the target genes. (**E**) In addition, EDCs can modulate the epigenetic profile of the cell (methyl mark and microRNA). (**F**) Finally, they can modulate the expression of enzymes involved in the catabolism of estrogens into hydrophilic compounds.

The most common mode of action of EDCs is binding to nuclear or membrane SRs (Figure 1C,D). EDCs can induce adverse biological effects by acting as agonists or antagonists of these receptors, but also by altering signal transduction initiated by these receptors or by interfering with the interaction of the receptors with partners regulating their transcriptional activities such as coregulators [53]. EDCs interact mainly with ERα/β, modifying the genomic and non-genomic pathways. The estrogen genomic pathway involves the binding of ERα/β to DNA sequences called estrogen response elements (EREs), or their binding to other transcription factors, resulting in the transcription of target genes. The estrogen non-genomic pathway involves membrane receptors (ERα, ER-α36 and the G protein-coupled receptor 30 (GPR30)), resulting in a rapid transduction of intracellular signals [18]. Some EDCs such as the dioxin TCDD can indirectly inhibit ERs through the aryl hydrocarbon receptor (AhR) [54]. This receptor was first identified as a detoxification receptor for xenobiotic aromatic hydrocarbons. Indeed, the binding of the aromatic hydrocarbon to AhR induces its translocation into the nucleus where it induces the expression of detoxification enzymes, leading to the elimination of aromatic hydrocarbons [55]. Since then, other roles for AhR have been acknowledged in carcinogenesis, cellular stress response and immunity. In addition, AhR interacts with the estrogen pathway by activating the transcription of estrogen-metabolizing enzymes, by competing for the recruitment of certain coregulators, or by inducing the degradation of ERα [56]. EDCs can also bind to AR and inhibit its action. For example, dichlorodiphenyldichloroethylene (DDE), a metabolite of DDT, has been shown to be a potent AR antagonist in male rat [57]. Unlike SRs, thyroid hormone receptors (TRs) appear to have very restrictive binding sites, only few EDCs have been described as agonists or antagonists [58]. For example, a PCB polychlorinated biphenyl (PCB) metabolite is known to have a direct agonistic effect on TRs [59].

EDCs can interact with SRs at low concentrations. This is intrinsic to the endocrine system, which is physiologically activated by low concentrations of circulating endogenous hormones. Indeed, physiological serum estradiol concentrations range from 10 to 900 pg/mL. Several reasons may explain how endogenous hormones act at low circulating concentrations: they have a very high affinity for their receptor, their dose-response relationship is non-monotonic, and the relationship between the number of bound receptors and the biological effect is also non-monotonic. Indeed, the occupation of few receptors can lead to a biological effect, leaving free receptors that can, for example, interact with EDCs. Similar to endogenous hormones, EDCs do not follow a monotonous dose-response relationship. It can be U-shaped (i.e., maximum response at the lowest and highest concentrations), inverted U-shaped (i.e., maximum response observed at intermediate doses), or without a particular shape (i.e., maximum response observed at several different doses). Various reasons may explain the non-monotonic dose-response relationship of EDCs. Indeed, EDCs can be cytotoxic and therefore no effects can be observed at high concentrations if the cells do not survive. In addition, EDCs may have different affinities for SRs. Finally, EDCs may also induce receptor degradation or desensitization [60].

Changes in endogenous hormone concentrations can have different consequences depending on the age of the individual. Similarly, risk factors have different effects depending on the time of exposure, during a window of susceptibility or not. An increase in BC incidence has been observed in Japanese atomic bomb survivors. This was the first time a window of susceptibility has been described for a risk factor of BC. Indeed, exposure to radiation during World War II bombing significantly increased the risk of BC in women exposed during childhood or adolescence [61]. The first BC risk-increasing EDC described as having a window of susceptibility is DES. Women exposed in utero to DES have an increased BC risk after 40 years of age, but not in younger women [62]. Since then, other EDCs have been described as having susceptibility windows. For instance, the effect of TCDD may vary according to the target organ as well as the moment of exposure [63]. Indeed, animal studies have shown that prenatal exposure to TCDD increased susceptibility to carcinogen-induced breast tumor formation, while exposure during pregnancy delayed breast tumor formation. However, only a few studies have considered specific exposures during windows of susceptibility [13].

Classical in vivo toxicology studies rely on tests based on the linearity of the dose-response effect to assess the no observable adverse effect level (NOAEL), the lowest observed adverse effect level (LOAEL), or the acceptable daily intake (ADI) of a substance [64]. Therefore, these classical pharmaco-toxicity studies are not always applicable to EDCs. Indeed, two different EDCs or one EDC and an endogenous hormone can bind simultaneously to a receptor and induce a synergistic effect. This mechanism, known as positive cooperativity, could be responsible for the “cocktail” effect of EDCs, which means that the adverse effect of a mixture of EDCs is greater than the sum of the negative effects of each EDC alone [60]. Indeed, a study on the activation of human ERα in recombinant yeast showed that a mixture of 8 xenoestrogens resulted in the activation of ERα, whereas each EDCs alone at the same concentrations had no effect [65]. Another study highlighted that exposure in utero and then by breastfeeding to 3 anti-androgens resulted in nipple malformation (inverted nipple) in Sprague-Dawley rats, whereas they had no effect when administered alone at the same doses [66]. Another study in which Sprague-Dawley rats were exposed in utero to 5 anti-androgenic phthalates alone or in combination confirmed their synergistic effect [67]. Finally, an interesting study showed that low doses of a synthetic estrogen and a pesticide synergistically activated the pregnane X receptor (PXR). The authors pointed out that the two together have a better affinity than when they are alone. The simultaneous binding to PXR stabilizes it, explaining PXR synergistic activation. This mechanism could explain the non-linear dose-response or the cocktail effects of certain EDCs [68].

#### 3.2.2. Modification of the Level of Bioavailable Endogenous Hormones

EDCs can also act on endogenous hormones by modulating their synthesis, transport, distribution, and clearance. Their synthesis is physiologically regulated by autocrine (hormones act on the cell that synthesized them), paracrine (hormones act on cells close to the cell that synthesized them), and endocrine (hormones act on cells distant from the cell that synthesized them) feedback mechanisms [53].

Steroid biosynthesis takes place in the adrenal cortex, gonads and placenta. These three organs de novo synthesize cholesterol from plasma lipoproteins, and the cholesterol is then transformed into different hormones such as progesterone, cortisol, testosterone and estradiol by two major families of enzymes, CYPs and hydroxysteroid dehydrogenases. In addition, hormone production can occur locally in certain peripheral tissues such as adipose tissue (including breast tissue) that produce estrogens from androgens [69]. Among the enzymes involved in estrogen synthesis, aromatase, encoded by the CYP19 gene, transforms androgens into estrogens. Dysregulation of aromatase expression will therefore have important repercussions on the various biological processes involving the androgen and estrogen hormones [70]. For example, some EDCs, such as neonicotinoid pesticides, can induce an increase in aromatase expression, leading to an increase in circulating estrogen concentrations (Figure 1A) [71]. Thyroxine prohormone (T4) is synthesized in the thyroid gland and then metabolized to the active hormone triiodothyronine (T3) in different ways [72]. Some EDCs such as perchlorate can inhibit the synthesis of thyroid hormones by preventing the absorption of iodine by thyroid cells [73]. In addition, BPA may interfere with the metabolism of thyroid hormones by reducing the activity of Type 1 iodothyronine deiodinase (DIO1), the enzyme that catalyzes the conversion of T4 to T3 [74].

Steroid hormones are lipophilic compounds, and their plasma transport is via specific proteins such as albumin, sex hormone-binding globulin (SHBG) and cortisol binding globulin (CBG). These proteins regulate the free fraction of steroid hormones in plasma, and thus their ability to access their target cells [75]. EDCs are also hydrophobic and may compete with endogenous hormones for binding to these transport proteins. This competition may alter the free fraction and bioavailability of endogenous hormones, and thus impact their target cells. Indeed, a study of 125 chemicals showed that more than 60% of them bind to SHBG, the major androgen and estrogen transport protein (Figure 1B) [76].

Steroid hormones are metabolized by enzymes that make them hydrophilic and inactive so they can be excreted by the kidneys. EDCs can also alter the inactivation and clearance of these steroid hormones. For example, the hormone responsible for estrogen sulfatation, estrogen sulphotransferase, can be inhibited by pesticides, thus increasing the concentrations of bioavailable estrogen by reducing its clearance (Figure 1F) [77].

#### 3.2.3. Alteration of the Cell Epigenome

Steroid hormones can modify epigenetic markers in DNA and histones as well as the expression of non-coding RNAs (ncRNAs), a common property with EDCs (Figure 1E) [53]. Different mechanisms may explain the impact of EDCs on the epigenome, including modulation of the expression of proteins involved in DNA modifications, post-translational modifications, as well as alteration of the expression of ncRNAs [78].

DNA methyltransferases (DNMTs) are involved in epigenetic regulation by methylating DNA. DNMT1 is involved in the maintenance of DNA methylation patterns, and the DNMT3 family is responsible for de novo methylation of the genome. The level of DNA methylation directly influences gene expression, as hypermethylated promoters are not transcribed [79]. These different enzymes of the epigenome can be regulated by endogenous hormones or by EDCs. For example, a histological study showed that DNMT1 and DNMT3a expression significantly decreased in the human endometrium during the mid-secretory phase. This study also showed in vitro that treatment with estradiol and medroxyprogesterone acetate induced a decrease in the expression of DNMT3a and DNMT3b in human endometrial stromal cells [80]. The same year, a study exposing Fischer rats in utero to a pesticide showed a hypermethylation of promoters encoding ERα/β correlated with increased expression of DNMT3b. Since DNMT3b is responsible for de novo methylation of the genome, fetal exposure to certain EDCs modifying its expression can alter methylation patterns in adults, and thus modify the expression of hyper- or hypo-methylated genes [81].

Among ncRNAs, microRNAs (miRNAs) regulate the translation of messenger RNAs (mRNAs) and their stability. In humans, about one-third of genes are thought to be regulated by more than 1000 miRNAs. They are therefore involved in many biological functions such as differentiation, proliferation and cell death, but also in the development of diseases such as cancer. Each miRNA potentially has about 100 mRNA targets, although targeting all mRNAs simultaneously is only possible if the miRNA level is high [82]. Cells exposed to a particular hormone or EDC may exhibit changes in the expression of certain miRNAs, a phenomenon called miRNA signature. A study showed that treatment of ERα+ breast and ovarian cancer cells with estradiol induced a decrease in the expression of several miRNAs (miR-181a, -21, -181b, -26a, -26b, -200c, - 27b, and -23b). This study also showed that 7 of these miRNAs were involved in estrogen-dependent cell growth [83]. Similarly, certain EDCs can modulate miRNA expression. Indeed, treatment of ERα+ BC cells with DDT or BPA induced a specific miRNA signature different from that induced by estrogen [84]. Since miRNAs regulate different biological processes, the induction of a miRNA signature following exposure to EDCs can have a significant impact on the fate of the exposed cells.

## 4. Case Studies of Endocrine Disrupting Chemicals Linked with Increased Risk of Breast Cancer

More than 1000 chemical agents have been evaluated by the IARC, and 121 of them have been classified as Group 1 “carcinogenic to humans” [50]. In vitro, in vivo, clinical and epidemiological studies required to associate an effect with a substance are either difficult to perform or to interpret (limited extrapolation of pre-clinical data) for EDCs. For epidemiological studies, it is very often case-control studies measuring the serum concentration of an EDC at the time of diagnosis of a disease. These studies are not suitable because, in addition to synergistic and cocktail effects, EDCs and their metabolites generally have various modes of action. Because of the possible long latency between exposure to EDCs and diagnosis, these case-control studies lead to results that are often not reproducible. A few prospective studies that measured exposure to EDCs several years before diagnosis of the disease have obtained more consistent results. As described above, exposure to radiation during childhood or adolescence increases the risk of developing BC [85]. The IARC classified ionizing radiation as Group 1 “carcinogenic to humans” for some cancer including BC in women [86]. In utero exposure to DES also increases the risk of developing BC [62], and the IARC classified it as Group 1 “carcinogenic to humans” [87].

Here four different EDCs will be discussed in detail: DES, DDT, dioxins, in particular TCDD and BPA. These EDCs have all been linked to the occurrence of BC and in vitro, in vivo, and epidemiological evidence is accumulating. They each have different histories and modes of action, as well as specific regulations, which illustrates the heterogeneity of EDCs.

### 4.1. Endocrine Disrupting Chemicals and Medicine: Diethylstilbestrol in Pregnant Women

In 1938, a team of Oxford researchers published a paper describing several stilbene derivatives with estrogenic activity, as part of a project to find easily synthesizable estradiol-like compounds. Among the derivatives described, DES was the most estrogenic [88]. Indeed, DES is 5 times more potent than estradiol, the most potent natural estrogen in mammals [89]. Clinical trials carried out to assess the impact of DES in pregnant women showed a decrease in miscarriages, premature births and cases of pre-eclampsia [90,91]. As a result of these studies, DES was marketed in the USA and then in other countries for the prevention of miscarriage, and also for the reduction of prematurity and pregnancy-related hemorrhages. The most common galenic form is the tablet, but it has also been on the market as an injectable solution, suppository and cream [92].

However, the control cohort was not randomly selected, and it was an open-label study [90,91]. Moreover, growing evidence of deleterious effects, in particular cancer development (see below) has since accumulated.

The evaluation of the number of women who have taken DES is difficult in part because of its many galenic forms. The majority of prescriptions were made in the USA between the 1940s and 1970s, where it is estimated that 5 to 10 million people were exposed to DES during pregnancy or in utero [93]. DES has also been widely used in Europe, where approximately 300,000 and 200,000 people have been exposed in the United Kingdom and France respectively [94]. In addition to its medical use, DES was used in animal farming to accelerate weight gain in animals from 1954 in the USA [95]. Less than 5-years later, more than half of the cattle farms in the USA were supplemented with DES.

#### 4.1.1. Diethylstilbestrol, a Synthetic Estrogen Inducing Significant Epigenetic Changes

In view of its strong estrogenic capacities, numerous in vivo studies in rodents have been conducted to evaluate the toxic and carcinogenic potential of DES shortly after its synthesis. Among these studies, some have highlighted the capacity of DES to induce reproductive and mammary abnormalities.

As previously mentioned, DES binds to ERα with an affinity 5 times greater than estradiol [89]. A first study on chronic exposure to DES in 3-rats strains showed the development of numerous pathological lesions including mammary gland cancers of all histological types [96]. Another study in which mice were chronically exposed to DES also showed the formation of mammary adenocarcinomas [97]. Furthermore, subcutaneous injection of 5 µg DES/kg body weight during the first 5 days postpartum results in stimulation of pituitary prolactin secretion. Increased prolactin levels have been correlated with hyperplasia of the mammary gland ducts [98]. The sensitivity of the mammary gland to DES was investigated in female mice that were injected with DES subcutaneously for the first 5 days of life. The effect of DES on different mouse tissues was evaluated and showed that the ovarian and mammary glands are between 10 and 100 times more sensitive to neonatal DES exposure than vaginal, uterine and adrenal tissues [99]. Another study in which 0.1 µg DES/kg body weight was injected subcutaneously during the first 5 days postpartum in mice showed mammary gland malformations similar to those observed 14 years earlier by Nagasawa with a 50-fold higher dose. This study reported hyperplasia of the mammary gland ducts, but also an increase in the number of TEBs and a decrease in the number of lobules, characteristic of a delay in the development of mammary glands [100].

Other studies have shown that exposure to DES in utero has an impact on the mammary gland of the offspring. Injection of DES into pregnant rats on days 15 and 18 of pregnancy resulted in dose-dependent mammary tumor formation in the offspring. Indeed, the injection of 4 µg DES/kg body weight led to the development of mammary tumors in 5 of 33 (15%) rats exposed in utero, but only in 1 of 45 (2%) for an injection of 0.4 µg [101]. A more recent study investigated the impact of in utero exposure to DES on BC risk (single oral exposure to a potent carcinogen, DMBA). Prenatal exposure to DES induced a significant increase in the number of female rats with benign and malignant proliferative lesions in the mammary gland. These results suggest that in utero exposure to DES could induce endocrine disorders and promote the induction of mammary carcinomas [85].

A transcriptomic analysis performed lately on the TEBs of Sprague-Dawley rats exposed to DES by a single subcutaneous injection 24 h after birth revealed that at day 35, a dose of 1 µg/kg body weight induced a change in the expression of 381 genes (181 over-expressed and 200 under-expressed compared to the control), while a dose of 100 µg/kg body weight induced the dysregulation of only 109 genes (35 over-expressed and 74 under-expressed compared to control). On day 49, the 1 µg dose induced a change in the expression of only 85 genes (49 over-expressed and 36 under-expressed compared to the control), and the 100 µg dose induced dysregulations of 65 genes (29 over-expressed and 36 under-expressed compared to the control). Among them, some genes were related to mammary gland differentiation and development, which could explain mammary gland malformations as well as tumor development [102]. In addition, intraperitoneal injection of 10 µg DES/kg body weight in pregnant mice resulted in a doubling of the expression of the Enhancer of Zeste Homolog 2 (EZH2), a histone methyltransferase, in the mammary cells of the offspring. The increased level of EZH2 is correlated with an increase in histone H3 lysine 27 tri-methylation (a residue specifically methylated by EZH2) [103]. Interestingly, immunohistochemical analysis of human breast tissue linked elevated expression of EZH2 in healthy tissue with an increased risk of tumor development. EZH2 is therefore a potential marker for the in vivo detection of preneoplastic breast lesions [104]. Recently, a study on mice exposed in utero to DES showed an alteration of the stroma around the mammary gland. Indeed, an intraperitoneal injection of 100 µg DES/kg body weight per day between the 9th and 18th days of pregnancy led to an increase in the rigidity of the mammary gland, probably due to an increase in collagen [105].

#### 4.1.2. Diethylstilbestrol during Pregnancy: Three Generations Impacted

In the 1960s, 7 cases of vaginal cancer were diagnosed in women between the ages of 15 and 22 at the Vincent Memorial Hospital in Boston. Since no diagnosis had ever been made in this age group and the 7 cases were diagnosed within 3 years, a retrospective study of the patients and their families was conducted. This study highlighted the use of DES in the mothers of these women during pregnancy. Nevertheless, DES was prescribed to 675 other patients in the hospital during this period and only 7 cases of vaginal cancer were diagnosed [106]. A few years later, a larger study confirmed an increased risk of vaginal cancer with in utero exposure to DES [107]. The diagnosis of vaginal cancer in several women exposed in utero to DES led to the follow-up of all persons exposed to DES in many countries. Five major cohorts including the National Cooperative Diethylstilbestrol Adenosis Project (DESAD) cohort, the Women’s Health Study cohort, the Mayo Clinic cohort, Dieckmann’s clinical study conducted at the University of Chicago (1951–1952), and Horne’s study conducted in a private clinic in Massachusetts were analyzed. The main studies are summarized in Table 2.

The effect of DES was first evaluated in mothers exposed during pregnancy, known as “DES mothers”. A first study published in 1978 compared the risk of BC between the cohort of 693 mothers who had participated in Dieckmann’s clinical study and 668 control mothers. This study showed a non-significant increase in the number of BC in the exposed women. However, this study was conducted soon after exposure to DES relative to the time it generally takes for the effects of EDCs to become clinically evident. Although no information is given on age at diagnosis in the study, it is possible that the majority of the cohort was younger than the median age of BC diagnosis since only a maximum of 25 years elapsed between the time of DES exposure and the study [108]. A study of 2885 DES mothers and 2816 controls found that DES exposure during pregnancy increased the risk of BC by 40–50% compared to unexposed women. Interestingly, the increase was statistically significant for women exposed 30 years prior the study. This study had a longer follow-up (up to 40 years after DES exposure), a larger cohort, and an auto-questionnaire that took into account other risk factors in the women of both cohorts [109]. Another study of 2590 DES mothers and 2471 controls investigated the effect of DES more than 40 years after exposure. After adjustment for various risk factors (age, age at menarche, age at first pregnancy, history of miscarriage), DES exposure during pregnancy increased the risk of BC by 47% in women of 60 years of age or more. This study has the same strengths as the previous study, with an even longer follow-up (beyond 40 years) [110]. Finally, a study involving 2434 DES mothers and 2402 controls from the Dieckmann and Mayo Clinic cohorts confirmed that women exposed to DES have an increased risk of BC, independent of other risk factors [111].

The effect of the drug was then evaluated in the children of DES mothers (i.e., those exposed in utero) called “DES daughters or sons”. In DES sons, an increased risk of genital malformations such as hypospadias (malformation of the urethra) or cryptorchidism were observed [119]. In addition to clear cell vaginal adenocarcinomas, several studies have reported an increase in cervical and uterine malformations, as well as problems during pregnancy (miscarriage, ectopic pregnancy, pre-eclampsia, premature birth, perinatal death), and infertility in DES daughters [120]. Concerning cancer risk, a first study of 3650 DES daughters versus 1202 control girls found no increased risk for all types of cancers including BC. This study was conducted on a large cohort of DES girls from the DESAD, Dieckmann and Horne cohorts, and different variables were taken into account such as year of birth, level of education, age at menarche, at menopause and at first birth, parity, oral contraceptive or HRT use, and family history of breast or ovarian cancer. Nevertheless, the average age of women was only 38 years, which is much younger than the median age of diagnosis of BC (around 67 years) [112]. Since this first study, others were conducted on large cohorts and showed an association between in utero exposure to DES and BC risk. A study including 3812 DES daughters and 1637 unexposed girls from different cohorts (DESAD, Women’s Health Study, Dieckmann and Horne) found a significant increase in BC risk in DES daughters but with age adjustment only. The increased risk of BC is greater in DES daughters than in the mothers. Indeed, women under the age of 40 had no increased risk, whereas the risk increased by more than 90% in women over 40 years of age. This risk is almost 4-times higher in postmenopausal women over the age of 50. Interestingly, the risk differed according to the characteristics of the tumor, as there was an increased risk only for ERα+ tumors. These results highlight the importance of long-term follow-up of women exposed to EDCs. This study took into account many risk factors, making it a statistically powerful multivariate analysis (year of birth, marital status, level of education, tabaco consumption (mother and daughter), oral contraceptive and HRT use, family history of BC, BMI and birth weight, age at menarche and menopause, parity, age at first birth, and number of mammograms in the last 5 years). This study also took into account gestational age at first exposure to DES as well as dose (low or high) [113]. One year later, a study regrouping 3813 DES daughters and 1642 controls from the same 4 cohorts and also taking into account numerous BC risk factors and gestational age at first DES exposure and dose, confirmed the increased risk in women over 40 years of age only. Indeed, DES daughters over 40 years of age had a BC risk increased by 83%, whereas no increase was found in younger women [62]. In 2011, a study combining the DESAD, Women’s Health Study and Dieckmann cohorts of 3796 DES daughters and 1659 controls also found a 40% increased risk of BC in women over 40 years of age in women exposed in utero to DES compared to control. Interestingly, this study also found an increase in adverse health outcomes such as infertility, spontaneous abortion, preterm delivery and pre-eclampsia [114]. More recently, a study analyzed the risk of BC in 4822 DES daughters compared to 2083 unexposed women from the DESAD, Women’s Health Study, Dieckmann and Horne cohorts. This study confirmed again an increased risk in DES daughters after age 40, but the increase in risk was less significant compared to data from the 2007 Troisi study. Indeed, only women between 40 and 49 years of age had a significant increase in BC risk of 33%. This study took into account many risk factors for BC as well as gestational age at first DES exposure and dose [115].

A French study published in 2015 on 3436 DES daughters and 3256 controls also found a doubling of BC risk in DES daughters. Indeed, the risk was increased for all DES girls by 88%. When year of birth, employment, age at menarche, number of pregnancies and births, age at first birth, and infertility treatment were taken into account, the risk doubled. BC risk also changed with DES dose: the increase was 63% for a low dose and 216% for a high dose. Nevertheless, only 25% of the women in this study had a medical certificate of exposure proving in utero exposure to DES [116]. 

Not all recently published studies find an increased risk of BC. Indeed, the Prospective Dutch study conducted on a cohort of 12,091 DES daughters found no increase in BC risk. Although this study was conducted on a large number of women and the median age at the end of the study was 44 years, in utero exposure to DES was medically confirmed in only 12% of subjects. Moreover, cancers diagnosed before the study were not taken into account [117].

Recently, a study of 796 children of women exposed in utero to DES, called “DES granddaughters or grandsons”, and 469 controls was published. The third generation presented an increase in genital malformations and other health problems similar to those of DES daughters or sons. Thereby, the impact of exposure of pregnant women to DES appears to be multigenerational, affecting the outcome of the third generation (e.g., hypospadias, miscarriage, ectopic pregnancy, premature birth). However, studies on the multigenerational effect of DES on adult diseases such as cancer are limited because the third generation is still young (average age 24 years in this study). Although this study was conducted on a relatively small cohort and statistical power was limited, it highlights the multigenerational impact of DES [118].

#### 4.1.3. Indication and Use of Diethylstilbestrol after the Tragedy

As previously mentioned, it was the diagnosis of vaginal cancers in DES daughters that led to the withdrawal of DES from the market in 1972 in the USA, and 6 years later in Europe [106]. DES is the first EDC described as having a window of susceptibility. Women exposed in utero to DES have an increased risk of BC after 40 years of age, but not in younger women [62]. Its use in animal farming was banned in 1979, but fodder was contaminated at least 8 years after the ban [95]. In addition to the teratogenic and carcinogenic effects observed, a double-blinded clinical trial conducted on a correctly selected cohorts did not show a reduction in the incidence of pregnancy complications in treated women [121].

The IARC have classified DES as Group 1 “carcinogenic to humans” [87]. In the USA, it was withdrawn from the market in all its forms and for all its uses as early as 1972 [122]. France was the only country in which DES remained on the market until 2018 for the treatment of prostate cancer [123].

### 4.2. Massive Use of Dichlorodiphenyltrichloroethane: Awareness of Environmental Pollution by Toxic Endocrine Disruptor Chemicals

DDT is an insecticide discovered in 1939 by the Swiss chemist Paul Hermann Müller, a discovery for which he was awarded the Nobel Prize for Medicine in 1948. As early as 1941, DDT was used against the Colorado potato beetle, a pest that was destroying European crops. During World War II, DDT was used as an anti-louse by the German army, and its anti-malarial properties were used by the USA and German armies. It was after the end of World War II that DDT was authorized for the civilian population in the USA, which led to its massive use until 1959. As early as the 1940s, the question of the toxicity of DDT on the environment and human health arose [124]. It was Rachel Carson’s book, Silent Spring, that opened the debate publicly [125]. DDT can persist in the environment for up to 15 years and also bioaccumulates in the food chain [126]. When an individual absorbs DDT, it is metabolized to DDE and dichlorodiphenyldichloroethane (DDD). DDT is used as a marker of recent or active exposure while DDE indicates past exposure [127].

#### 4.2.1. The Dual Mode of Action of Dichlorodiphenyltrichloroethane, Both Estrogenic Agonist and Androgenic Antagonist

Numerous in vitro studies have investigated the underlying mechanisms of DDT pathogenicity. Its estrogenic properties were discovered by screening in MCF-7 cells (luminal type; ERα+, PR+, HER2−) [128]. A few years later, a study on ERα+ BC cells showed that 0.3 µM DDT stimulates the cell entry into S-phase. In the same way as endogenous estrogens, DDT induces the transcription of genes involved in cell cycle regulation via the binding to ERα [129]. Following these findings, a study compared genotypes of MCF-7 cells exposed to estradiol with those exposed to DDT. The authors found that treatment with 10 µM DDT or 1 nM estradiol altered the expression of 13 genes involved in the signaling of BC. Some genes were regulated differently by DDT, including the vascular endothelial growth factor-A (VEGF-A). The increase in VEGF-A expression by DDT was independent of ERα. DDT directly activated the hypoxia-inducible factor-1 (HIF-1) response element of VEGF-A promoter. This study also showed that DDT increases the activity of p38 MAP kinase, which in turn increases the transcriptional activation of CBP, a general transcriptional coactivator [130].

In addition, DDE was rapidly identified as a potent AR antagonist. Indeed, an in vitro study on rat prostate cells showed that DDE competitively bound to AR and induced an anti-androgenic effect similar to the synthetic anti-androgen hydroxyflutamide [57]. Recently, a study showed that DDE induced a dose-response increase in the proliferation of CAMA-1 (luminal type; ERα+, PR+, HER2−) and MCF-7-AR1 (MCF-7 cells overexpressing AR) BC cell lines in the presence of physiological concentrations of estrogens and androgens. The androgen signaling pathway inhibits the growth of hormone-responsive BC cells. The antagonistic action of DDE on this pathway overcomes BC cells growth inhibition, and thus induces BC progression [131].

Various studies have shown that DDT modulates the expression of enzymes involved in estrogen metabolism and catabolism. Indeed, a study on BC cells showed that 1 µM DDT induced an increase in aromatase expression, independently of the estrogen signaling pathway. The authors also showed that the increase in aromatase was correlated with an increase in cyclooxygenase 2 (COX-2) and prostaglandins [132]. Another recent study highlighted that 5 µM DDT regulated the transcription of several genes involved in different biological processes such as oxidative stress, inflammation and escape from the immune system. Among them, the expression of the gene coding for CYP1A1, a phase I enzyme involved in estrogen catabolism, was significantly decreased. This decrease was correlated with the decrease in AhR, a known inducer of CYP1A1 [133]. Interestingly, AhR is involved in cell proliferation and cell survival signaling pathways. In breast cells (tumorigenic and non-tumorigenic), AhR was shown to interact with the nuclear factor-κB (NF-κB) transcription factor involved in cell survival [134].

In addition, treatment of ERα+ BC cells with 10 µM DDT induced a particular miRNA signature distinct from that induced by estrogen. For example, it induced an increase in miR-21 which negatively regulates the translation of maspine and the Programmed Cell Death Protein 4 (PDCD4) mRNAs. These proteins are tumor suppressor proteins, the down regulation of which leads to increased cell proliferation and migration [84].

In addition to these in vitro studies, a few in vivo studies on BC have been performed in rodents. The first one, conducted in 1968, showed that treatment with DDT produced the same estrogenic effects as estradiol in rats (140 mg DDT/kg body weight), chicken (136 mg DDT/kg body weight) and quail (190 mg DDT/kg body weight) [135]. A few years later, another study in Wistar-Furth rats injected with human mammary adenocarcinoma cells and treated daily with 50 mg DDT/kg body weight showed that DDT promotes the growth of estrogen-sensitive mammary tumors [136]. A study in Sprague-Dawley rats showed that exposure to 100 mg DDT/kg body weight by subcutaneous injection after birth resulted in increased proliferation of breast cells, which promoted maturation of TEBs into intralobular ducts [137]. Another study showed that subcutaneous injection of 50 mg DDT/kg body weight led to clastogenic effects on cell ploidy (aneuploidy, polyploidy) in the mammary gland of Sprague-Dawley rats [138]. From an epigenetic point of view, several studies have confirmed in vivo the observations made in vitro. Indeed, intraperitoneal exposure of female rats to 75 mg DDT/kg body weight induced an alteration in the expression of oncogenic miRNAs (miR-221, -222, -205, -126a and -429), as well as their target genes involved in hormonal carcinogenesis, including aromatase [139]. Another study also confirmed in vivo the increase in miR-21 in female rats treated intraperitoneally with 50 mg DDT/kg body weight [140]. Intrestingly, DDT has shown a transgenerational epigenetics effect. Indeed, exposure to 25 mg DDT/kg body weight in pregnant rats resulted in altered DNA methylation and ncRNAs over three generations [141].

Even though DDT has been banned in several developed countries since the 1970s, many women born in the years of massive DDT use are still alive. In addition, DDT can persist up to 15 years in the environment after having been used and can also bioaccumulate in the food chain. Today, DDT can still be absorbed through inhalation or contaminated food [126].

#### 4.2.2. Prepubertal Exposure to Dichlorodiphenyltrichloroethane and Breast Cancer Occurrence

There are now more than 500 epidemiological studies investigating the impact of exposure to DDT or DDE on BC incidence, and some of them are meta-analyses. The majority of these studies retrospectively look for an association between DDT exposure and BC occurrence by measuring serum concentrations at the time of diagnosis. However, few are prospective. Due to differences in methods (choice of cohort, period of exposure and other risk factors taken into account, design, methods, etc.), these studies do not have homogeneous results [142]. The main studies are summarized in Table 3.

The largest study is the Child Health and Development Study (CHDS), which includes serum samples from approximately 20,500 pregnant women collected between 1959 and 1966 and serum samples of their children, followed for 50 years. This cohort was used to study the impact of DDT exposure on BC under different circumstances. Extensive socio-economic and demographic information was collected from women and spouses (place of birth, age, occupation, household income, etc.); maternal history (number of pregnancies, tabaco use, medical history and drug use, blood pressure, etc.); and newborn information (sex, plurality, weight, height, gestational age, etc.) [151]. The first study investigating BC risk in the CHSD separated the 129 pairs of case-control samples according to the patient’s age at the time of exposure to investigate the correlation between DDT and DDE serum concentrations and BC occurrence. In this way, the authors showed that elevated DDT serum concentrations were associated with a 5-fold increase in BC risk only in women born after 1931. These women were therefore less than 14-years-old when DDT was used by the civilian population in the USA, and about 20 years-old at the time of its peak use. Women born before, and therefore older at the time of exposure, did not show an increased risk of BC. The strengths of this study are the consideration of the existence of windows of susceptibility and the collection of samples during the period of exposure to DDT. However, this study did not take into account the other risk factors described above between postpartum serum sampling and diagnosis [143]. Another study based on the CHDS cohort (103 cases and 315 controls followed over 54 years) found that elevated maternal DDT serum concentrations during pregnancy increased the occurrence of BC in the daughter by a factor of 4 to 5. In addition, elevated maternal serum DDT was positively associated with advanced stage at diagnosis and the development of HER2+ tumors in the daughter, independently of maternal overweight and maternal BC history [144]. A more recent study on a CHDS sub-cohort compared 146 women diagnosed with BC between 50 and 54 years of age to 422 controls. This study showed that exposure to DDT increased the risk of BC in women first exposed after 4 years of age and not before, and when BC is diagnosed before the age of 54. Combining the information from this study with that of Cohn (2007), it can be assumed that the window of susceptibility of the mammary gland for DDT is during childhood and puberty (between 3 and 13 years of age) [145]. The CHDS cohort and the information derived from it underline the difficulty of studying EDCs because the delay between exposure and its consequences extends over several decades. Moreover, these results confirm in humans what had already been demonstrated in rodent models. For some, this indicates that in vivo rodent studies should be better taken into consideration when developing public health policies based on the precautionary principle [152].

The Long Island Breast Cancer Study Project (LIBCSP) was conducted to determine whether the risk of BC in women is associated with exposure to certain persistent organic pollutants (POPs) including DDT. For this study, blood samples from approximately 1500 women diagnosed with BC between 1996 and 1997 compared to around 1550 controls, environmental samples, and self-report questionnaires were collected [153]. One of the LIBCSP-based studies analyzed the occurrence of BC in women who reported seeing truck spreaders during DDT use when they were young. Women with hormone-dependent BC have a 44% greater risk of having ever seen truck spreaders and thus of having had acute exposure to DDT, than women with other BC subtypes. The risk of developing BC was increased by about 30% for women born before 1945 who reported seeing truck spreaders. The strength of this study is that the values were adjusted according to age, ethnicity, number of pregnancies, age at menarche, breastfeeding, BMI, tobacco and alcohol consumption and oral contraceptive use. Nevertheless, this is a retrospective study and the blood samples were collected 25 years after the restriction of DDT use in the USA. Similarly, there is a risk of memory bias since the questionnaires use recollections of distant childhood events [146]. This study contradicts another more recent study based on the Sister Study cohort, which includes more than 2000 women aged 35–74 years who have not developed BC but whose sisters have been diagnosed. This prospective study found no significant increase in BC risk in women exposed to truck or airplane spreader prior to the ban on DDT [147]. Other studies have found no correlation between high serum DDT concentrations and BC risk, such as the French study based on the CECILE cohort of 695 cases compared to 1055 controls [148] or the Japanese study conducted on 403 pairs of case-control samples [149]. The major limitation of these studies is that they were performed on serum samples collected long after the massive use of DDT. A meta-analysis of 40 studies highlights that, overall, the correlation between exposure to DDT and BC is not statistically significant. According to the authors of this meta-analysis, the quality of the evaluation may be a cause of heterogeneity. Indeed, the methodologies used display differences in specificity and sensitivity [150]. Another meta-analysis of 35 DDE studies reached the same conclusions [142].

Many studies have looked for a correlation between exposure to DDT and the occurrence of BC. Although the results are heterogeneous, large cohort studies, which took into account the specificities of EDCs and in particular windows of susceptibility, have shown a strong correlation with childhood exposure and the occurrence of BC.

#### 4.2.3. Partial Ban of Dichlorodiphenyltrichloroethane and Current Regulations

Following the discovery of DDT’s deleterious effects and the associated occurrence of several diseases, DDT was banned in many countries between 1970 and 1980, including the USA and European countries [154,155]. DDT is currently considered to be a POP, i.e., it is inherently toxic, accumulates in the food chain and in the environment, and can travel long distances from its source [156]. In addition DDT has been classified as Group 2A “probably carcinogenic to humans” by the IARC [157].

Following the Stockholm Convention in 2001, the strict regulation of DDT in many countries has slightly reduced its global production and use worldwide [158]. Despite this, DDT and DDE are still detectable in various human biological samples such as serum, milk and hair. Indeed, a large meta-analysis of more than 400 studies from 60 countries confirmed the rapid decline of DDE between 1970 and 2001. This study also highlighted that the ban on DDT did not completely eliminate circulating DDE, with significant regional differences [159]. Indeed, the WHO still recommends the use of DDT as an antimalarial agent in endemic areas [160]. For example, DDT is used to control vector-borne diseases including malaria in Brazil. As a result, high concentrations of DDT have been found in the breast milk of women living in villages in the Madeira River basin in the Amazon. Thus, 8.7% of children had a daily intake of DDT exceeding the estimated ADI of 0.01 mg DDT/kg body weight [161,162]. The same observation was made in the breast milk of women living in malaria-endemic villages in South Africa [163].

The ban on DDT following Rachel Carson’s book was widely controversial at the time and seen as anti-capitalist. For some, it led to the return of malaria to areas where it was eradicated and had a negative impact on agricultural production [164]. Nevertheless, the use of other pesticides confirmed Carson’s fears about the negative consequences of excessive use of organochlorine and organophosphorus pesticides, particularly the resistance of pest species and the negative impact on wildlife and human health. In addition, the strengthening of pesticide regulation has encouraged the production of pesticides that are less harmful to human health and the environment [165].

### 4.3. Industrial Accident and Release of Toxic Dioxins into the Environment: Current Exposure and Risks

Dioxins are a class of related chemicals, which include polychlorinated dibenzo-para-dioxins (PCDDs) and polychlorinated dibenzofurans (PCDFs). Their characteristics and toxicity depend on the number and location of chlorines. Of the 210 existing dioxins and dioxin-like chemicals, only 17 are toxic. The most toxic dioxin is TCDD, which is used as a toxic equivalent (TEQ) [166]. The major source of dioxins is human activity as they are by-products of many industrial processes such as chemical manufacturing of herbicides and insecticides, combustion and metal smelting. The only natural sources of dioxins are forest fires and volcanic eruptions [167]. Dioxins are POPs and bioaccumulate in the environment. Human exposure occurs mainly through the ingestion of contaminated food, but also through polluted air [168].

The USA military contaminated the environment with TCDD by using large quantities of Agent Orange during, among others, the Vietnam War (1955–1975). Agent Orange is a 50/50 mixture of two herbicides (2,4,5-trichlorophenoxyacetic acid and 2,4-dichlorophenoxyacetic acid) that was spread using aircraft spreaders. The use of Agent Orange was part of Operation Ranch Hand, which was designed to destroy crops and forest cover for enemies. TCDD is a by-product of the manufacture of Agent Orange, and its presence in small amounts could not be detected initially. In the 1970s, new techniques made it possible to detect its presence, which led to the end of Operation Ranch Hand and the massive use of Agent Orange [169].

#### 4.3.1. 2,3,7,8-tetrachlorodibenzo-p-dioxin, a Potent Agonist of the Aryl Hydrocarbon Receptor

The molecular modes of action of TCDD are well known today. In 1973, a study in rats exposed orally to 5 or 25 µg TCDD/kg body weight showed a potent induction of the expression of enzymes involved in the metabolism of xenobiotics [170]. A few years later, a study provided evidence of a direct relationship between the TCDD-AhR complex and the induction of the expression of CYP1, a phase I enzyme of metabolism [171]. In addition, AhR activation by 10 nM TCDD in both tumorigenic (MCF-7) and non-tumorigenic (MCF-10A) breast cells led to the inhibition of the apoptotic response induced by radiotherapy (UV radiation) and chemotherapy (doxorubicin, lapatinib and paclitaxel) [172]. Interestingly, TCDD effects differ depending on the dose. Indeed, a study on M13SV1 cells (human breast luminal epithelial) treated with 0.01, 0.1, 1, 10 or 100 nM TCDD showed an inverted U-shaped dose-response effect on proliferation and gene expression including CYP1A1 [173].

In vitro studies have investigated the impact of TCDD on BC cells and found an anti-estrogenic effect. A study on T47D cells (luminal type; ERα+, PR+, HER2−) showed that exposure to 0.1 nM TCDD induced inhibition of cell proliferation stimulated by the transforming growth factor α (TGFα). A higher concentration of 10 nM TCDD also induced inhibition of cell proliferation stimulated by estradiol [174]. A study on chronic exposure to TCDD on MCF-7 cells at 1 nM showed complete inhibition of ERα expression, reversible rapidly after treatment cessation. Furthermore, this study confirmed that chronic exposure to TCDD induced the expression of the enzymes CYP1A1 and CYP1A2 involved in estrogen metabolism [175]. Another study confirmed the anti-estrogenic effects of TCDD at 100 nM and showed that the binding of TCDD to AhR modulated the activation of the Breast cancer type 1 susceptibility protein (BRCA1) promoter via ERα. Indeed, transcription of BRCA1 requires binding of the estradiol-ERα complex and activated AhR to the proximal promoter. The presence of the TCDD-AhR complex inhibits the activation of the BRCA1 promoter in MCF-7 cells [176]. 

In addition to TCDD binding to AhR and its anti-estrogenic action, a study has highlighted the induction of progesterone signaling pathways though PR. Indeed, TCDD induced MCF-7 cell proliferation by increasing the activity of proteins that regulate cell cycle progression while decreasing those that inhibit the cycle. As a result, 50 nM TCDD induced MCF-7 cell proliferation. Inhibition of AR led to the disappearance of these effects, suggesting the involvement of AR in the development of TCDD-induced BC [177].

Numerous in vivo studies have also investigated the effects of exposure to TCDD on the mammary glands of rodents, showing diverging effects at different life stages. A first study showed a delay in sexual maturation in rats exposed in utero (maternal gavage with 1 µg TCDD/kg body weight on day 15 after conception) compared to control rats. Prenatal exposure to TCDD also induced an increase in the number of TEBs and a decrease in the number of lobules 50 days after birth. This is characteristic of delayed mammary gland development, thus prenatal exposure to TCDD increases the susceptibility to BC [178]. Using the same method, another study showed that exposure in utero and by breastfeeding resulted in an increase in the number of TEBs and a decrease in the number of lobules in rats, correlated with an increase in the level of ERα expression (mRNA and protein). This study also showed that the mammary gland retains its ability to differentiate in response to the stimulation of exogenous estrogens. Therefore, perinatal exposure to TCDD increases susceptibility to BC [179]. A year later, another study used the same method of exposure but fed mothers at different times. Offspring were exposed either in utero on the 15th or 20th day after conception or by breastfeeding on the 1st, 3rd, 5th or 10th day after birth. Interestingly, only in utero exposure on the 15th day after conception induced a malformation of the mammary gland [180]. A more recent study on a carcinogen-induced rat mammary cancer model confirmed that prenatal exposure to TCDD increases the susceptibility to BC [181]. Several studies have since investigated in vivo the impact of TCDD on mammary epithelial cell function. A study on rat mammary tissue exposed in utero to TCDD showed a 40% reduction in BRCA1 and an 80% reduction in CYP1A1 mRNA, confirming observations made in vitro on BC cells [182].

AhR-expressing and non-expressing mice exposed to 100 µg TCDD/kg body weight by intraperitoneal injection presented different gene expression profiles. This study highlighted the complexity of the cellular response to TCDD exposure since approximately 50 genes were differentially expressed by a factor of 3 or more. Among the 28 genes induced was CYP1A1, but also genes involved in cell adhesion, cell proliferation and carcinogenesis, cell stress response, inflammatory response, and immune response. As for the 23 repressed genes, some were involved in cell metabolism, growth, cell cycle or tumor growth inhibition [183]. Interestingly, pregnant mice exposed to 1 µg TCDD/kg body weight exhibited a reduction in the expression of E-cadherin, a protein involved in cell adhesion. In addition, levels of the milk protein β-casein and the signal transducer and activator of transcription 5 (STAT5), a regulator of its expression, were also decreased [184]. Another study orally exposing pregnant rats to TCDD showed decreased maternal production of prolactin, a pituitary hormone essential for breastfeeding, and decreased milk production. The study also revealed a disturbance in the mother’s grooming and nursing behavior, as well as the offspring’s body weight and short-term memory. These different events could be mediated by AhR as they were not observed in AhR-knockout females [185].

Interestingly, TCDD had different effects depending on the hormonal status of the tissue. Indeed, a study on mice with constitutively active AhR injected with 3 µg TCDD/kg body weight showed that TCDD induced estrogenic effects in the absence of estrogen, but anti-estrogenic effects in the presence of estrogen. This could be explained by the ERα-AhR crosstalk observed in vitro [186].

#### 4.3.2. Exposure to Toxic Dioxins and Breast Cancer Risk: Heterogeneous Results

Agent Orange was used extensively in Vietnam by the USA military (approximately 76 million liters between 1961 and 1971). To date, studies on the exposed population have been conducted on cohorts of Vietnamese or USA veterans but have not assessed the risk of BC [187]. The majority of the published studies on TCDD come from cohorts exposed following industrial incidents. Several cases of dioxin contamination have been described such as household disinfectants produced by Monsanto in the 1930s, road pavement in California in the 1970s, and fodder in Belgium in the late 1990s [166]. In 1976, an accident at a chemical plant in Seveso, Italy resulted in the contamination of 18 km^2^ with 15 to 30 kg TCDD [188]. Since 1967, a chemical plant in Chapayevsk, Russia has been linked to high dioxin concentrations in soil, air, drinking water, cow’s milk, but also in human breast milk and serum [189]. The main studies are summarized in Table 4.

Several studies have been conducted on the Seveso cohort. A total of 5544 people resided in the area close to the explosion (zone A and zone B), including 2721 women [192]. The Seveso Women’s Health Study (SWHS) included women who were under 40 years of age at the time of the accident and for whom a serum sample was taken within 4 years of the explosion and stored. This corresponds to 1271 of the 2721 (47%) women living around the plant at the time of the explosion. Of the 1271 eligible, 981 (77%) women participated. Of these women, 21 were diagnosed with cancer, including 15 with BC (1.5% of the cohort). Using TCDD plasma concentrations as a variable, the authors found a dose-response relationship between TCDD plasma concentration and BC risk. Indeed, for a 10-fold increase in TCDD plasma concentration there was a doubling of the risk. This study is prospective, and several risk factors were taken into account to adjust the risk (number of pregnancies, breastfeeding, smoking, age at the time of exposure). Nevertheless, the main limitation of this study is the low number of cancer cases. In addition, the study population was young (only 26% of the study population was over 50 years of age at the time of analysis). Since the median age of diagnosis of BC is around 67 years of age, additional cases may have developed after this study [190]. A second follow-up was conducted between 2008 and 2009 on 833 of the 981 women in the initial cohort, 66 of whom had cancer, 33 of whom had BC (4% of the cohort). Using TCDD plasma concentrations as a variable as in the previous study, the authors found a dose-response trend but not significant [191].

Another epidemiological study on the Seveso disaster reviewed 2122 medical records of people diagnosed with cancer between 1992 and 1996, including 287 with BC (12.7%). The authors investigated whether the patients lived near the plant at the time of the explosion (zone A and zone B) or further away (zone R). This study showed that women living in the most contaminated zone (zone A) had a slight but significant increase in the risk of developing BC 15 years after the accident. No increase was observed in zones B and R. This study is prospective, several risk factors were taken into account to adjust the risk (lifestyle, occupation, diet and leisure activities), and the number of cases is important. Nevertheless, as with the previous two studies, this study was conducted soon after exposure to TCDD with regards to the time it generally takes for the effects of EDCs to become clinically evident [192].

The Chapayevsk chemical plant, in operation since 1967, employed about half of the city’s population in the 1990s. A study compared the incidence and mortality of BC among women in Chapayevsk with those of Russian women in general. In 1998, there was approximately a 50% increase in deaths from BC in Chapayevsk compared to the national average. There was also a doubling of the risk of developing BC among these women compared to the national average. These observations correlated with high concentrations of dioxin in the environment as well as in serum and breast milk. In contrast to the Seveso disaster studies, this study links chronic exposure to high levels of dioxins to increased incidence and mortality of BC in women. The main focus of this study is the comparison of dioxin levels in the environment (soil) and in food (vegetables, water, milk) with dioxin levels in serum and breast milk. Furthermore, the sampling was done at a time when the chemical plant was operating at only 20% of its capacity [189].

However, other studies investigating dioxin exposure and BC risk did not report a significant association. This is the case, for example, in a case-control study nested in the French E3N (*Etude Epidemiologique aupres de femmes de la Mutuelle Generale de l’Education Nationale*) cohort including 63,830 women. In this prospective study, the authors investigated dietary dioxin exposure and BC risk. Overall, there was no significant association between higher dietary dioxin exposure and BC risk. This study took into account many risk factors (age, parity, age at first birth, breastfeeding, menopausal status, HRT use, energy intake, BMI, tabaco and alcohol consumption and personal history of benign breast diseases) [193]. Another study on the French E3N cohort including 429 cases and 716 controls investigated BC risk and airborne dioxin exposure. This study found no significant association between higher airborne dioxin exposure and BC risk, although it did take into account many risk factors (age, age at diagnosis, BMI, level of education, tabaco and alcohol consumption, living space (rural or urban), oral contraceptive and HRT use, family history of BC, age at menarche and menopause, parity, breastfeeding, and number of mammograms) [194].

Nevertheless, a USA study based on 112,397 women from the Nurses’ Health Study II cohort found an association between airborne dioxin exposure and BC risk. Indeed, living less than 10 km from a municipal solid waste incinerator increased the risk of BC by 15%. The risk increased to 25% by living less than 5 km away. Interestingly, the risk did not vary according to the hormonal status of the tumor, nor the menopausal status. This study was prospective, and took into account many risk factors (age, family history of BC, menopausal status, age at menarche, parity, breastfeeding, oral contraceptive use, BMI, tabaco and alcohol consumption, diet, physical activity, income, marital status and region of residence) [195].

Furthermore, a recent meta-analysis of three studies did not support a statistically significant association between exposure to dioxins and the risk of BC. The observed inconsistency seems to be due to small-sampling size, inaccurate exposure assessments, and lack of historical exposure data able to estimate past exposures for decades before BC diagnosis [196]. In addition, to date, few epidemiological studies have investigated the suggested effect in animal studies of early-life exposure and BC risk later in life [13]. Indeed, one study compared serum levels of toxic dioxins in adolescents living in polluted or rural areas. This study highlighted that environmental exposure to toxic dioxins can impact sexual maturation and for example lead to delayed breast maturation [197]. A longitudinal study on the long-term effects of exposure to toxic dioxins on puberty confirmed this observation. The authors measured exposure via breast milk and serum in 33 children from birth to 14–19 years of age, and found that high exposure to toxic dioxins is correlated with delayed breast development [198]. In addition, the differences in the results of epidemiological studies may be explained by the non-linear dose-response relationship of TCDD and its different modes of action depending on the hormonal status of the tissues [174,178].

#### 4.3.3. Industrial Risk Management and Control of Dioxin Release

TCDD is classified as Group 1 “carcinogenic to humans” by the IARC [168]. The WHO sets the ADI at 1 to 4 pg dioxin TEQ/kg body weight [199]. In the USA, the FDA has been conducting programs to monitor dioxin levels in food since 1999 in order to reduce dietary exposure [200]. In other countries, regulations on the release of dioxins into the environment are stricter. This is the case in European countries where a directive was adopted in 1982 following the accident at the Seveso chemical plant in Italy. After being replaced and amended, the Seveso III directive of 2012 is currently in force. This directive provides a framework for industrial risks in Europe related to plants that handle hazardous substances. The Seveso directive requires manufacturers to identify risks and apply measures to prevent them [201,202]. Then in 2006, the European Commission set thresholds for maximum levels of dioxin in foodstuffs for the first time [203,204].

Since the 1990s, dioxin emission into the environment has been significantly reduced. This is mainly due to changes in technological processes and new regulations like the Stockholm convention in 2001. Nowadays, the main sources of dioxin emission arise from the production of electricity, iron and steel industries and non-ferrous metal production [205].

### 4.4. Bisphenol A: Difficulty in Tracing Exposure to a Synthetic Estrogen Ubiquitously Present in the Environment

BPA was first synthesized by the German chemist Zincke in 1905 [206]. Its estrogenic potency was discovered more than 30 years later at Oxford, during the project looking for synthetic estrogens which discovered DES. Indeed, this team showed the ability of BPA to block the menstrual cycle in rodents [207]. Two years later, the very potent DES was discovered, and BPA was abandoned [88]. Ten years later, the Swiss chemist Castan synthesized the first epoxy resin using BPA [208]. BPA became the main component of epoxy resins, and the global production of polycarbonate plastics is estimated at about 6.2 million tons in 2020, and is expected to increase reaching an estimated 7.2 million tons by 2027 [209]. In 2017, the EPA estimated that more than 450,000 of BPA are released into the environment each year [210]. Currently, BPA is found in many household products (bags, bottles, can/carton coatings, kitchen utensils, cosmetics, dental and medical products, paints, CD-ROMs, etc.). It can be released into the environment during its production, transport and disposal. Once in the environment, BPA can be incorporated into the soil by bacteria, fungi or algae [211]. Humans are mostly exposed by ingesting contaminated food or beverages. Indeed, the BPA contained in can/carton coatings leaches when they are old, heated, or in contact with high or low pH products. For example, the sterilization of cans induces the leaching of 80–100% of BPA into food [212]. In addition, one study reported that plasma BPA concentrations do not decrease rapidly during fasting. This suggests a non-dietary exposure and/or accumulation in adipose tissue to maintain serum BPA concentrations [213]. Indeed, humans are exposed daily and throughout their lives: more than 90% of the USA population over 6 years-old have detectable levels of BPA in their urine [214].

#### 4.4.1. Bisphenol A, a Synthetic Estrogen Similar to Diethylstilbestrol

The primary purpose of BPA was to be used as a synthetic estrogen. Since its structure is very close to that of DES, different scientists were interested in its in vitro and in vivo effects following the DES tragedy [88,207]. Although humans are exposed to very low doses, the characteristics of EDCs and in particular their non-monotonic dose-response curve quickly made BPA one of the most studied EDCs.

A study on MCF-7 BC cells confirmed the weak estrogenic effects of BPA. Indeed, the affinity of BPA for ERα was 3000 times lower than that of estradiol, and its proliferative potential was approximately 60,000 times lower [215]. A more recent study on the same cell line confirmed the pro-proliferative effect of 10 µM BPA, which is due to an increase in the expression of genes involved in cell cycle regulation (cyclin A and D, cyclin-dependent kinase 1 (CDK1) and 2). In addition, BPA modulates the p38/mitogen-activated protein kinases (MAPK) pathway [216]. Nanomolar concentrations of BPA were also reported to induce an increase in cancer cell motility in vitro [217]. A study on HeLa cervical cancer cells revealed another nuclear receptor, estrogen-related receptor-γ (ERRγ) activated by BPA with high affinity. ERRγ is an orphan nuclear receptor, the expression of which has been associates with poor patient prognosis in breast tumors. The role of this transcription factor, and the impact of its binding to BPA have not yet been fully elucidated [218]. More recently, two studies demonstrated that low BPA concentrations (in the nanomolar range) induced an increase in ERRγ-mediated cell proliferation and migration in ERα/β+ BC cells [219] and TNBC cells (ERα-, PR- and HER2-) [220]. BPA also stimulated the expression of homeobox B9 (HOXB9) similarly to estradiol. HOXB9 plays an important role in mammary gland development and is also associated with the development of BC [221]. The non-genomic pathway can also be activated by BPA. Indeed, a study on different BC cell lines showed that BPA induced a rapid activation of extracellular signal-regulated kinases 1 and 2 (ERK-1 and -2). This activation is independent of ERα, but dependent on the non-genomic pathway mediated by GPR30 [222]. A very recent study compared gene expression in different cell types exposed to 1 nM BPA for 30 days: MCF-7, SK-BR3 (HER2-enriched types; ERα−, PR−, HER2+) and MDA-MB-231 (TNBC types; ERα−, PR−, HER2−). The authors determined by mRNA sequencing that chronic exposure to BPA modulates the expression of genes involved in various biological functions. Interestingly, BPA modulated different biological functions in the three BC cell lines. Thus, BC subtypes seem to be impacted differently by EDCs [223].

In addition to its estrogenic action, a study has shown that BPA interacts with the AhR pathway. Indeed, 10 µM BPA decreased the expression of the Aryl hydrocarbon Receptor Nuclear Translocator 2 (ARNT2), a mandatory partner of AhR. ARNT2 is involved in a variety of physiological processes, and its deregulation could impact on BC pathogenesis and therapeutic responses [224]. BPA also showed anti-androgenic activities on mouse fibroblast cells. As the androgen signaling pathway inhibits the growth of hormone-responsive cells, BPA antagonistic on this pathway could induce hormone-responsive tumor progression [225].

Transcriptome studies have shown that MCF-7 cells exposed to 10 µM BPA have a distinct miRNA signature, which modulates ERα-controlled protein levels. Indeed, exposure to BPA led to a decrease in miR-15b and -27b, and an increase in miR-21, -138, -663 and -1915. As for DDT, the increase in miR-21 induced a decrease in maspine and PDCD4 levels [84].

In parallel, numerous in vivo studies have evaluated the effect of BPA on the development of the mammary gland and the occurrence of cancer. BPA was administered at high or low doses due to its likely non-monotonic dose-response relationship. In utero exposure to 25 µg BPA/kg body weight in mice induced a significant increase in the number of TEBs and of lobules 6 months after birth. These changes were associated with an a increase in secretory product in the alveoli, related to the altered expression of developmental genes [226]. In addition, exposure to the same dose increased the sensitivity of mammary glands to estradiol in ovariectomized mice [227]. Exposure of pregnant mice to 0.25 µg BPA/kg body weight significantly increased ductal area and fat pad maturation in exposed fetuses and induced a decrease in cell size and a delay in lumen formation [228]. A transcriptomic study revealed that mice exposed in utero to 0.25 µg BPA/kg body weight showed altered expression of genes involved in the pathways of adhesion, adipogenesis and apoptosis. These changes substantiated previously reported malformations of the mammary gland such as delayed lumen formation and increased fat pad maturation [229]. Recently, a study on mice exposed in utero to BPA exhibited an alteration of the stroma around the mammary gland. Indeed, an intraperitoneal injection of 25 µg BPA/kg body weight/day between the 9th and 18th day of pregnancy resulted in an alteration of the expression of 47 genes in the fibroblasts. These genes code for proteins involved in carcinogenesis and collagen fiber regulation. This alteration was associated with an increase in collagen deposition in adult female mice as well as a modification of their structure. Thus, exposure to BPA increases mammary gland collagen density [105].

Studies in rats have reported similar effects. Rats exposed to 25 µg BPA/kg body weight in utero showed hyperplasia of the mammary ducts, associated with signs of desmoplasia, characteristic of pre-neoplastic lesions [230]. In addition, rats exposed in utero to 2.5 µg BPA/kg body weight displayed ductal hyperplasia and in situ ERα+ breast carcinoma [231]. A further study revealed that exposure to 250 µg BPA/kg body weight induced specific methylation mark on more than 7000 DNA segments. This was associated with an increase in the expression of genes associated with cell cycle regulation such as cyclin-dependent kinase inhibitor 1c (CDKN1c). These events could contribute to the development of pre-neoplastic and neoplastic lesions that will arise in adulthood [232].

A study on rhesus monkeys exposed in utero to 400 µg BPA/kg body weight revealed an increase in TEBs and a complexification of the mammary epithelial tree. This dose, although higher than that used in rodent studies, allows to reach circulating levels of biologically active BPA (unconjugated BPA) close to levels measured in human serum (1 ng/mL) [233]. BPA therefore has a similar action on the development of mammary glands between rodents and primates, suggesting that it would not be without effect in humans.

#### 4.4.2. A Ubiquitous Presence Making Epidemiological Studies Difficult

As previously mentioned, BPA is a ubiquitous EDC. Several studies measured the concentrations of BPA in the environment. A meta-analysis of more than 500 studies reported BPA concentrations in different types of samples. In water, the concentration of BPA ranged from non-detectable to 370 µg/L in effluents and 56 µg/L in surface water. In municipal wastewater treatment plants, the concentration of BPA ranged from 10 to over 100,000 µg/kg dry weight. In soils, concentrations ranged from non-detectable to 1000 µg/kg. In indoor air, the maximum concentration of BPA was measured in a resin plant in China (more than 50,000 ng/m^3^), and the minimum concentration in residential and commercial buildings (less than 100 ng/m^3^. BPA has also been measured in different foodstuffs: between 0.2 and 13,000 ng/g in fish, and similar concentrations have been measured in amphibians, mollusks, shellfish, aquatic insects and seaweed [234]. At room temperature, water in plastic bottles contains between 0.2 and 0.3 mg BPA/L [235]. Another study measured BPA in thermal paper, and revealed that the one used for receipts is responsible for the transcutaneous exposure of 41 to 71 µg BPA/day in cashiers [236]. These BPA exposure levels are in the same order of magnitude as the concentrations used in in vivo studies in rodents, and may increase the risk of developing hormone-dependent cancers such as BC in human.

There have been no incidents of human exposure to high doses of BPA. Therefore, no epidemiological studies comparing a high exposure cohort with a low exposure cohort could be performed. Despite this, a few epidemiological studies have looked for an association between high levels of BPA and the risk of BC. All of these studies are summarized in Table 5.

The first epidemiological study on BPA and BC was the National Health and Nutrition Examination Survey (NHANES). This study analyzed the impact of exposure to BPA on the occurrence of different pathologies in almost 1500 people. High urinary concentration of BPA was not associated with an increased risk of BC, despite adjustment for age, ethnicity, tabaco consumption and BMI. Nevertheless, this study was retrospective, and all cancers were analyzed in a single category. In addition, there was no follow-up on the occurrence of cancer after sample collection, and cancer history was based on patient self-reporting [237].

In 2014, a Polish study found no association between exposure to BPA and BC risk in postmenopausal women. This retrospective study was conducted on urine samples from 575 case-control pairs [238].

Other studies reported an association between high concentrations of BPA in serum or urine and BC risk factors. A Korean study on serum BPA levels in 70 cases and 82 controls found an association between high serum concentration of BPA and nulliparity, a risk factor for BC, but not with an increased BC risk. This study was prospective: samples were collected between 1994 and 1997 and stored for around 10 years. However, it had several limitations: the cohort was small, the mean age at the time of the study was only 46 years, and there was little time between sampling and diagnosis [239].

Another USA study, the Wisconsin Breast Density Study, found a significant association between high BPA serum concentrations and another risk factor for BC, high breast density. This study was performed on 264 postmenopausal women and serum concentrations of BPA were adjusted for age, BMI and other risk factors. Nevertheless, this study was retrospective and conducted on a small cohort [240].

In 2018, a Chilean study, the Growth and Obesity Cohort Study, analyzed the relationship between breast density and urinary concentration of BPA before and at the end of puberty in 200 adolescent girls. This study found that girls with the lowest and highest urinary concentrations of BPA had a breast density at least 10% higher than girls with medium concentrations. Thus, this study shows that BPA has a U-shaped association with risk factor for BC, high breast density. The urinary concentrations were adjusted according to the age and BMI of the adolescents, but also according to the level of education of the mother [241]. As previously mentioned, a 5% increase in density has been correlated with a 5–10% increase in the risk of BC [34] (Table 1).

Given the wide variability in methodology, epidemiological studies conducted on a larger number of patients and taking into account the specificities of EDCs are necessary to obtain sufficient evidence and determine the degree of correlation between low-dose BPA exposure and BC. Nevertheless, the similarity between DES and BPA, and the different in vivo results obtained in rodents and primates encourage the application of the precautionary principle discussed during the Wingspread conference.

#### 4.4.3. The Precautionary Principle behind Bisphenol A Legislation in Some Countries

Numerous in vitro and in vivo studies have shown the effects of BPA at high and low concentrations. Interestingly, a meta-analysis highlighted that more than 90% of government-funded studies show that BPA has effects at low doses, while industry-funded studies show no effect [242]. 

Despite the accumulation of evidence, regulations vary considerably from one country to another. For example, in Europe, the ADI for BPA was set at 50 µg/kg body weight/day. In view of the impact of BPA at nanomolar levels in rodents, the ADI in Europe has been lowered to 4 µg/kg body weight/day. Since 2018, a new working group is evaluating recent data on BPA in order to update the scientific opinion on this substance [243]. In 2019, Europe classified BPA as an EDC [244]. In other countries such as the USA, BPA regulations are less strict. After a meta-analysis of more than 300 studies, the FDA concluded that BPA-containing plastic in contact with food does not pose a threat to human health [245].

Although BPA is banned in plastic in contact with food in some countries, it is still present in many of the products mentioned above. In addition, other molecules that can be used as alternatives to BPA (bisphenol AF, bisphenol AP, bisphenol B, bisphenol F, bisphenol S and bisphenol Z) do not appear to be any less estrogenic. Indeed, a study on BC cells showed that different BPA analogues induced the same gene expression profile as BPA. This observation needs to be validated by other in vitro and in vivo studies. Nevertheless, it underlines the importance of better understanding the deleterious effects of exposure to BPA alternatives, particularly on hormone-dependent pathologies [246].

## 5. Discussion

BC is a heterogeneous disease, both histologically and molecularly. In addition, the mammary gland develops throughout a woman’s lifetime, including periods particularly vulnerable to the formation of neoplastic lesions. The occurrence of BC is due to the interplay of many individual and environmental risk factors, some of which are preventable, such as exposure to harmful chemicals.

This review focused on four established EDCs described as associated with increased BC risk. Although they are now banned or strongly regulated in several countries, they represent pertinent models for other EDCs. Indeed, there is a lot of information available on their in vitro mechanisms of action that have been confirmed in vivo in rodents or primates. There are also a large number of epidemiological studies, often with heterogeneous results. This is partly due to the ubiquitous presence of EDCs at low concentrations, making it difficult to form unexposed control groups.

These four case studies shed light on the issues surrounding the understanding and regulation of EDCs. Indeed, the regulation of chemical substances is based on classical toxicology studies that establish the NOAEL and the LOAEL in order to determine an ADI. These different measurements are valid for molecules inducing a dose-dependent effect, which is often not the case for EDCs [60]. In addition, exposure to EDCs early in life appears to have a delaying effect on puberty during adolescence. Indeed, the delay in development of the mammary gland after prepubescent exposure to EDCs could have a long-term impact on the risk of developing BC [247]. Moreover, many EDCs are present at detectable levels in the environment. Their ubiquitous presence at low concentrations underscores the importance of structuring current research to increase knowledge about them. Indeed, the EPA estimates that the USA population is exposed daily to approximately 87,000 endocrine disrupting chemicals potentially causing an increased risk of various pathologies including cancers [248]. The main problem in regulating EDCs is the lack of reliable epidemiological data. Currently, few EDCs have been classified as human carcinogens by the IARC. Usually, the epidemiological data that led to this classification were obtained following a health catastrophe or the misuse of a molecule.

Members of the Endocrine Society published their recommendations for EDCs. They highlight the link between different pathologies, including hormone-dependent cancers in women, and different EDCs, including BPA and dioxins. The authors report that over the last 5 years, several publications have led to a better understanding of the mechanisms of action of EDCs, including non-monotonic dose-response relationships, low dose effects and vulnerability during certain exposure windows. In view of this new information, they recommend the development of international collaborations in order to study EDCs more effectively. They highlight the importance of educating and raising awareness among the future generations of researchers, chemists, physicians and public health experts. They insist on the importance of prioritizing EDCs in research funding in order to establish effective prevention. Finally, they advocate changing current regulations and in particular reducing the amount of evidence needed, recalling that absolute proof of harm or proof of safety is not possible [249].

Despite the awareness of the population and the public authorities, EDCs are still part of the daily life of many populations. Indeed, their limitation would have major economic consequences, since several targeted substances are used by large industries such as pesticides or are by-products of industrial processes such as dioxins. In 2016, a collective of 100 scientists denounced the industrial lobby in a column in the newspaper *Le Monde* [250]. In this article, scientists from different countries called on the international community to act against EDCs. For them, scientific results are manipulated when they go against commercial interests. This manipulation of science, deliberate according to the collective of scientists, prevents the precautionary principle mentioned during the Wingspread conference. According to them, this leads to a delay in preventive actions, sometimes with serious consequences for the health of populations and the environment.

A good example of industry interference in the regulation of EDCs is BPA. As previously mentioned, the results on the carcinogenicity of BPA are different depending on the source of funding for the studies [242]. In addition, following BPA classification as an EDC in Europe by the European Chemicals Agency, Plastics Europe, an association of plastics producers, has appealed to the European Court of Justice. On 19 September 2019 the European Court of Justice ruled in favor of the European Chemicals Agency and confirmed the classification of BPA as an EDC [244].

EDC pollution is recent, yet it spares no organism or environment. Moreover, it is likely that their long-term effects, especially their multigenerational and potential transgenerational effects remain underappreciated.

## Figures and Tables

**Table 1:** Common breast cancer (BC) risk factors.

Risk	Example	Impact	Refs
**Reproductive factors**	Age at menarche	BC risk decreases by 5% for each year without menstruation between 11 and 17 years of age	[29]
	Age at menopause	BC risk decreases by 3% for each year without being menopausal between 35 and 55 years of age	
	Age at first birth	BC risk increases by 3% before menopause and 5% after menopause for each year that first full-term pregnancy is delayed	[30]
	Parity	Each full-term pregnancy decreases BC risk by 3% before menopause and 12% after menopause	
	Breastfeeding	Breastfeeding decreases BC risk by 14% before menopause and 11% after menopause	[31]
**Exogenous hormones**	Combined hormonal replacement therapy (HRT)	BC risk increases by 60% for 1 to 4 years of use and by 108% for more than 5 years of combined HRT use	[10]
	Hormonal contraception	BC risk increases by 0.7% for each year of contraceptive use	[11]
**Anthropometric factors**	Body mass index (BMI)	The risk of postmenopausal BC increases by 40% for every 10-point increase in BMI	[32]
**Sex and age**	Sex	Less than 1% of BC develop in men	[33]
	Age	More than 70% of BC are diagnosed after 50 years of age	[2]
**Breast density and personal history of BC**	Breast density	A 5% increase in breast density increases BC risk by 5 to 10%	[34]
	Personal history	Surviving BC increases the risk of developing second primary BC by 74%	[35]
**Familial history of BC**	First-degree family history	One history of BC increases the risk by 77% Two or more histories of BC increase the risk by 250%	[36]
	*Breast cancer type 1 susceptibility protein* mutation	55% risk of developing BC after 70 years of age	[37]
	*Breast cancer type 2 susceptibility protein* mutation	47% risk of developing BC after 70 years of age	
**Lifestyle**	Diet	Consumption of 120 g per day of red meat increases BC risk by 11%	[38]
	Tobacco	BC risk increases by 0.5% for each year of smoking	[39]
	Alcohol	Every unit of alcohol (10 g of alcohol) drunk per day increases BC risk by 7%	[40]
	Physical activity	BC risk decreases by 18% with the practice of 1 to 3 h of physical activity per week and 21% for more than 7 h per week	[41]
**Occupation**	Night shift work	20 years or more of rotating nightshift work at baseline induce a 2-fold increase in BC risk 20 years or more of cumulative rotating night-shift work increases BC risk by 40%	[42]
**Exposure to radiation**	Hodgkin lymphoma radiation	29% risk of developing BC after 55 years of age for women who received chest radiation before 25 years of age	[43]

**Table 2:** Overview of epidemiological studies on diethylstilbestrol (DES) and breast cancer (BC) risk.

Author (Year)	Study Years	Country	Design	Cases/Controls	Exposure Assessment	Results
Bibbo (1978) [108]	1976–1977	USA	Prospective	693/668	Participants in the 1951 clinical study	No significant increase in BC risk in DES mothers
Greenberg (1984) [109]	1981	USA	Prospective	2885/2816	Obstetric records	Significant increase in BC risk for DES mothers exposed more than 30 years prior the study (RR = 2.5; 95% CI: 1.1–5.8)
Colton (1993) [110]	1986–1989	USA	Prospective	2590/2471	Obstetric records	Significant increase in BC risk for DES mothers after 60 years of age (RR = 1.47; 95% CI: 1.02–2.13)
Titus-Ernstoff (2001) [111]	1992–1994	USA	Prospective	2434/2402	Obstetric records	Significant increase in BC risk for DES mothers exposed less than 40 years prior the study (RR = 1.27; 95% CI: 1.07–1.52)
Hatch (1998) [112]	1978–1994	USA	Prospective	3650/1202	Obstetric records	No significant increase in BC risk in DES daughters (RR = 1.18; 95% CI: 0.56–2.49)
Palmer (2006) [113]	1978–2003	USA	Prospective	3812/1637	Obstetric records	DES daughters have a significantly increased BC risk after 40 years of age (RR = 1.91; 95% CI: 1.09–3.33) and after 50 years of age (RR = 3.00; 95% CI: 1.01–8.98)
Troisi (2007) [62]	1978–2001	USA	Prospective	3813/1642	Obstetric records	DES daughters have a significantly increased BC risk after 40 years of age (RR = 1.83; 95% CI: 1.1–3.2)
Hoover (2011) [114]	1975–2001	USA	Prospective	3796/1659	Obstetric records	DES daughters have a significantly increased BC risk after 40 years of age (HR = 1.82; 95% CI: 1.04–3.18)
Troisi (2019) [115]	1994–2011	USA	Prospective	4822/2083	Obstetric records	DES daughters have a significantly increased BC risk between 40 and 49 years of age (RR = 1.33; 95% CI: 1.05–1.66)
Tournaire (2015) [116]	2013	France	Prospective	3436/3256	Self-report or medical records	DES daughters have a significantly increased BC risk (RR = 2.10; 95% CI: 1.60–2.76) but risk varies with low (RR = 1.63; 95% CI: 0.87–3.08) or high (RR = 2.16; 95% CI: 1.18–3.96) DES dose
Verloop (2010) [117]	1992–2008	Netherlands	Prospective	12,091 participants	Self-report or medical records	No significantly increase in BC risk in DES daughters (RR = 1.05; 95% CI: 0.90–1.23)
Titus (2019) [118]	2001–2012	USA	Prospective	796/469	Obstetric records	DES granddaughters have genital malformations and other health problems similar to those of DES daughters

**Table 3:** Overview of epidemiological studies on dichlorodiphenyltrichloroethane (DDT) or dichlorodiphenyldichloroethylene (DDE) and breast cancer (BC) risk.

Author (Year)	Study Years	Country	Design	Cases/Controls	Exposure Assessment	Results
Cohn (2007) [143]	2000–2001	USA	Prospective	129/129	Serum (1959–1967)	High DDT serum concentrations are associated with a significant increase in BC risk in women born after 1931 (OR = 5.4; 95% CI: 1.7–17.1)
Cohn (2015) [144]	2010–2013	USA	Prospective	103/315	Serum (1959–1967)	High DDT serum concentrations in mothers are associated with a significant increase in BC risk (OR = 3.7; 95% CI: 1.5–9.0); advanced stage at diagnosis (OR = 4.6; 95% CI: 1.3–16.5); and Human Epidermal Growth Factor Receptor 2 + (HER2+) tumors in daughters (OR = 2.1; 95% CI: 1.0–4.8)
Cohn (2019) [145]	1970–2010	USA	Prospective	146/422	Serum (1959–1967)	Exposure to DDT after 4 years of age significantly increases the risk of BC diagnosed before the age of 54 (OR = 3.70; 95% CI: 1.22–11.26)
White (2013) [146]	NA	USA	Retrospective	1508/1556	Residential exposure by questionnaire	Women with hormone-dependent BC have a significantly greater risk of having ever seen spreaders (OR = 1.44; 95% CI: 1.08–1.93) Women with Estrogen Receptor + (ER+) or Progesterone Receptor + (PR+) BC have a significantly increased odds of ever seeing a fogger truck (OR = 1.33; 95% CI: 1.11–1.59)
Niehoff (2016) [147]	2003–2009	USA + Puerto Rico	Prospective	2134 participants	Residential exposure by questionnaire	No significant association between having ever seen a spreader before DDT ban and BC risk (HR = 1.3; 95% CI: 0.92–1.7)
Bachelet (2019) [148]	NA	France	Retrospective	695/1055	Serum (2005–2007)	No significant association between high DDE serum concentrations and BC risk (OR = 0.93; 95% CI: 0.73–1.18)
Itoh (2009) [149]	NA	Japan	Retrospective	403/403	Serum (2001–2005)	No significant association between high DDT serum concentrations and BC risk (OR = 0.58; 95% CI: 0.27–1.25)
Ingber (2013) [150]	2012	Multi-centric	Meta-analysis	40 DDT or DDE studies	Serum	No significant association between BC risk and high serum concentrations of DDT (OR = 1.02; 95% CI: 0.92–1.13) or DDE (OR = 1.04; 95% CI: 0.94–1.15)
Park (2014) [142]	2012	Multi-centric	Meta-analysis	35 DDE studies	Serum	No significant association between high DDE serum concentrations and BC risk (OR = 1.03; 95% CI: 0.95–1.12)

**Table 4:** Overview of epidemiological studies on 2,3,7,8-tetrachlorodibenzo-p-dioxin (TCDD) and breast cancer (BC) risk.

Author (Year)	Study Years	Country	Design	Cases/Controls	Exposure Assessment	Results
Warner (2002) [190]	1996–1998	Italy	Prospective	981 participants	Serum (1976–1981)	A 10-fold increase in TCDD plasma concentrations was associated with an increase in BC risk (HR = 2.1; 95% CI: 1.0–4.6)
Warner (2011) [191]	1996–2008	Italy	Prospective	833 participants	Serum (1976–1981)	No association between high TCDD serum concentrations and BC risk (HR = 1.44; 95% CI: 0.89–2.33)
Pesatori (2009) [192]	2006-2009-	Italy	Prospective	2122 participants	Medical records (1992–1996)	Living near the chemical plant during the accident significantly increases BC risk (RR = 2.57; 95% CI: 1.07–6.20)
Revich (2001) [189]	1997–1998	Russia	Prospective	14 participants	Human milk and serum (1997–1998)	BC incidence and mortality are doubled in Chapayevsk compared to the national average
Danjou (2015) [193]	1993–2008	France	Prospective	63,830 participants	Dietary exposure	No significant association between higher dietary dioxin exposure and BC risk (HR = 1.00; 95% CI: 0.96–1.05)
Danjou (2019) [194]	1993–2008	France	Prospective	429/716	Airborne exposure	No significant association between higher estimated airborne dioxin exposure and BC risk (OR = 1.124; 95% CI: 0.693–1.824)
VoPham (2020) [195]	1989–2013	USA	Prospective	112,397 participants	Airborne exposure	Living less than 10 km from a municipal solid waste incinerator significantly increases BC risk (HR = 1.15; 95% CI: 1.03–1.28) The risk increases again by living less than 5 km away (HR = 1.25; 95% CI: 1.04–1.52)
Xu (206) [196]	2015	Multi-centric	Meta-analysis	3 studies	Various	No significant association between higher TCDD exposure and BC risk (RR = 0.99; 95% CI: 0.93–1.06)

**Table 5:** Overview of epidemiological studies on bisphenol A (BPA) and breast cancer (BC) risk.

Author (Year)	Study Years	Country	Design	Cases/Controls	Exposure Assessment	Results
Lang (2008) [237]	2003–2004	USA	Retrospective	1455 participants	Urine (2003–2004)	No significant association between high urinary BPA levels and cancer risk (including BC) (OR = 1.12; 95% CI: 0.85–1.48)
Trabert (2014) [238]	2000–2003	Poland	Retrospective	575/575	Urine (2000–2003)	No significant association between high urinary BPA levels and postmenopausal BC risk (OR = 1.09; 95% CI: 0.73–1.63)
Yang (2009) [239]	2004–2007	Korea	Prospective	70/82	Serum (1994–1997)	Significant association between high serum BPA levels and nulliparity (*p* < 0.05) No significant association between BPA levels and BC risk (*p* = 0.42)
Sprague (2013) [240]	2008–2009	USA	Retrospective	264 participants	Serum (2008–2009)	Significant association between high serum BPA levels and high breast density (*p* = 0.01)
Binder (2018) [241]	2006	Chile	Prospective	200 participants	Urine (2006)	Significant association between lower and higher urine BPA levels and high breast density (*p* < 0.01)

## Abbreviations

ADI	Acceptable Daily Intake
AhR	Aryl hydrocarbon Receptor
AR	Androgen Receptor
BC	Breast Cancer
BMI	Body Mass Index
BPA	Bisphenol A
*BRCA1/2*	Breast Cancer type 1/2 susceptibility protein
DDE	Dichlorodiphenyldichloroethylene
DDT	Dichlorodiphenyltrichloroethane
DES	Diethylstilbestrol or stilbestrol
DNMTs	DNA methyltransferases
EDCs	Endocrine Disrupting Chemicals
EPA	Environmental Protection Agency
ERα/β	Estrogen Receptor α/β
ERE	Estrogen Response Elements
ERRγ	Estrogen-Related Receptor γ
FDA	Food and Drug Administration
GPR30	G Protein-coupled Receptor 30
HER2	Human Epidermal Growth Factor Receptor 2
HRT	Hormone Replacement Therapy
IARC	International Agency for Research on Cancer
LOAEL	Lowest Observed Adverse Effect Level
miRNA	microRNA
mRNA	messenger RNA
NOAEL	No Observable Adverse Effect Level
PDCD4	Programmed Cell Death Protein 4
POP	Persistent Organic Pollutant
PR	Progesterone Receptor
SR	Steroid Receptor
TCDD	2,3,7,8-tetrachlorodibenzo-p-dioxin
TEB	Terminal End Buds
TEQ	Toxic Equivalent
TNBC	Triple Negative Breast Cancer
WHO	World Health Organization
